# Advancements in Hyaluronic Acid Effect in Alveolar Ridge Preservation: A Narrative Review

**DOI:** 10.3390/diagnostics15020137

**Published:** 2025-01-08

**Authors:** Paul Andrei Nistor, Andreea Cândea, Iulia Cristina Micu, Andrada Soancă, Carmen Silvia Caloian, Alina Bârdea, Alexandra Roman

**Affiliations:** Department of Periodontology, Faculty of Dental Medicine, Iuliu Hatieganu University of Medicine and Pharmacy, 400012 Cluj-Napoca, Romania; paulnistor91@gmail.com (P.A.N.); andreea_candea@yahoo.com (A.C.); andrapopovici@gmail.com (A.S.); alina.stanomir@yahoo.com (A.B.); veve_alexandra@yahoo.com (A.R.)

**Keywords:** hyaluronic acid, tooth extraction, wound healing, tooth socket, alveolar bone loss

## Abstract

**Background/Objectives**: Tooth extraction induces significant alveolar ridge dimensional changes and soft tissue modifications, often leading to challenges in implant placement or conventional prosthetic rehabilitation. Alveolar Ridge Preservation (ARP) strategies aim to mitigate post-extraction resorption of the alveolar ridge, enhancing both the quality and quantity of bone and soft tissue during healing. Hyaluronic acid (HYA) has emerged as a promising biological agent for ARP due to its osteoinductive, antimicrobial, and anti-inflammatory properties. However, the effects of HYA in ARP remain inconsistently reported. This study aims to assess current clinical and preclinical evidence regarding the biological effects of HYA and its application in ARP. Additionally, it evaluates HYA’s impact—alone or in combination with other products—on hard and soft tissue dimensional changes, early wound healing, and implant success rates. **Methods**: A comprehensive electronic literature search was conducted, and studies meeting the inclusion criteria were critically evaluated. Relevant data were extracted from the final selection of articles. **Results**: Thirteen publications were evaluated. Some studies reported a significantly improved newly formed bone following ARP with intra-socket HYA application as a single approach (*p* = 0.004). Combining HYA with a bone graft and a free palatal graft resulted in significantly greater amounts of newly formed and mature bone, reduced clinical bone width changes, lower radiographic crestal bone loss (*p* < 0.01), and diminished radiological volumetric and linear bone resorption (*p* = 0.018). Short-term follow-up data indicated improved soft tissue healing associated with HYA-based ARP. While HYA appears to have a protective effect on ridge dimensional changes in ARP, other studies reported no significant differences in radiographic bone dimensional changes or soft tissue improvement. **Conclusions**: The addition of HYA to bone grafts may enhance some ARP outcomes. However, the variability in outcomes and methodologies across the evaluated studies precludes drawing definitive clinical conclusions. Further robust research is needed to clarify HYA’s role in ARP. With respect to clinical significance enhancing the understanding of ARP management strategies and their effects on post-extraction sockets empowers clinicians to make more informed decisions. The knowledge of HYA effects facilitates the selection of personalized ARP approaches tailored to optimize outcomes for subsequent interventions.

## 1. Introduction

### 1.1. Ridge Alterations After Tooth Extraction

Following tooth extraction, the healing process leads to substantial three-dimensional resorption of the socket walls, causing changes in both the hard and soft tissues, as well as a shift in the contour of the alveolar ridge prior to extraction [1]. The loss of alveolar bone can negatively affect the aesthetic and functional results of dental implant placement and prosthetic restoration [2,3].

Bone resorption following extraction does not occur uniformly. The amount of new bone that forms within the extraction socket and the degree of volumetric reduction in the alveolar ridge can differ not only between individuals but also across various sockets within the same patient [2,4,5]. Several human studies revealed relatively large dimensional changes during the first 3–6 months after extractions, followed by gradual long-term bone reduction with an estimated annual bone contour loss of 0.5–1%, occurring predominantly on the buccal side of the ridge [2,6]. According to a systematic review of re-entry studies, vertical changes in the first six months after extraction can reach 11–22%, with reductions of up to −1.24 ± 0.11 mm at the buccal side, and smaller changes of 0.84 ± 0.62 mm mesially and 0.80 ± 0.71 mm distally. Horizontal reduction is often more severe, ranging from 29% to 63% (−3.79 ± 0.23 mm) [2]. These processes result in a shift of the anterior alveolar ridge in a more lingual or palatal direction, leading to a deficit in the contour in the labial region [1,7].

Bone resorption patterns also differ between the mandible and maxilla, with the mandible typically experiencing greater bone loss [6]. However, the clinical relevance of this difference remains unclear [8]. A recent systematic review [9] noted a mean horizontal bone loss of 3.61 mm (95% CI: 3.24–3.98) in the posterior region and 2.54 mm (95% CI: 1.97–3.11) in the anterior region. In terms of vertical bone loss, the buccal bone diminished by 1.46 mm in the molar region and 1.65 mm in the anterior region [9].

A more recent study [10] synthesized data from multiple systematic reviews [2,4,9,11,12,13,14,15,16,17,18] and observed greater height reduction of the buccal and lingual aspects of the ridge compared to mesial and distal sides following extraction. This is likely because the proximal bone is preserved by the periodontal ligaments of adjacent teeth [19]. Furthermore, the reduction in crestal width decreases as the distance from the alveolar crest increases [2].

Soft tissue changes in post-extraction sites have not been as extensively studied, and recent research on this topic remains limited [20].

### 1.2. Factors Influencing Post-Extraction Dimensional Changes

The degree of post-extraction ridge resorption varies significantly between patients, with differences in the rate, duration, and extent of bone loss [21]. The extent of changes in both bone and soft tissue dimensions following tooth extraction is influenced by a variety of general and local factors, making it difficult to predict accurately [2,4,5]. Factors such as the surgical trauma from the extraction itself [19], pre-existing bone loss, chronic infections [22], the extraction of multiple adjacent teeth [19,23] as well as patient characteristics like sex, age, and ethnic background, can accelerate bone resorption post-extraction. However, it remains uncertain whether minimally invasive techniques provide significant advantages for Alveolar Ridge Preservation (ARP) procedures [24]. Systemic conditions such as diabetes [25], vascular disease, malnutrition [26], immunodeficiency, osteoporosis [27], renal diseases [28], endocrine disorders, and smoking [29] can impair or delay the normal physiological and metabolic processes involved in socket wound healing.

The periodontal phenotype may also play a crucial role in post-extraction bone remodelling [19]. A thicker periodontal phenotype tends to better preserve the pre-extraction ridge contour, as the thicker socket wall and gingival tissue provide additional protection. The presence of both alveolar lamellar bone and bundle bone can help prevent complete bone loss after extraction. At 8 weeks post-extraction, the mean bone loss in height at the mid-buccal aspect of maxillary single-rooted teeth was 7.5 mm for sockets with a bone thickness of less than 1.0 mm, compared to only 1.1 mm for those with a bone thickness of 1.0 mm or more. Increased bone resorption was notably more common in areas with a thinner periodontal phenotype, such as in the incisor and canine regions, compared to the thicker phenotypes found in premolar areas [30]. These findings are particularly important in the context of the high aesthetic demands associated with the maxillary anterior region.

### 1.3. Alveolar Ridge Preservation

ARP refers to procedures aimed at minimizing or preventing the post-extraction resorption of the alveolar ridge while encouraging bone formation within the socket [13,14,31]. It also seeks to improve both the quality and quantity of soft tissue during socket healing [8]. The ideal objectives of any ARP procedure include the following: (1) limiting dimensional changes of the alveolar ridge post-extraction, maintaining the ridge contour to facilitate implant placement, and ensuring proper tooth-supported prosthetics; (2) promoting new bone formation within the socket at a level conducive to the osseointegration of a dental implant; (3) supporting soft tissue healing at the socket opening, ensuring compatibility with aesthetic and functional prosthetic outcomes; and (4) minimizing the need for future soft and bone ridge reconstruction procedures [6,8,20,32].

When comparing ARP to unassisted socket healing, there is less reduction in bone loss of 0.16–1.72 mm in vertical mid-buccal bone height and 1.61–1.99 mm less reduction in horizontal bone width with ARP [33,34].

The current literature does not indicate that implants placed in preserved sockets are more prone to peri-implant diseases than those placed in intact bone. In fact, a substantial body of research from recent studies and systematic reviews supports the efficacy of ARP as a reliable method to ensure successful implant placement and maintain aesthetic harmony in the oral cavity [35].

Although significant progress has been made, there is no consensus on the best ARP technique for various clinical scenarios [8,36]. No evidence has conclusively proven the superiority of one material or technique over others for ARP, and the choice of technique remains at the clinician’s discretion [37].

Several techniques for ARP have been suggested, using bone grafting materials, collagen membranes, matrices, and biological products [2,8,38]. These techniques are often selected based on factors such as implant placement feasibility, the quality and quantity of mucosal coverage in the region, the remaining buccal bone height, and expected implant success rates [6]. Studies have indicated that using absorbable collagen membranes may better preserve the alveolar ridge width when compared to xenogeneic or allogeneic grafts alone [34,39]. Additionally, the use of membranes [13,40] or biologics [41,42] shows promising results in achieving effective ARP.

Some studies have suggested that certain ARP techniques and materials can reduce soft tissue dimensional changes after extraction [43,44,45,46], while other research does not support this view [19,47]. Most ridge preservation studies have focused on fresh extraction sockets, but in clinical practice, extractions are often performed due to chronic pathologies as a result of endodontic or periodontal lesions. For ARP in these cases, it is important to use biomaterials with enhanced biological properties capable of addressing bone alterations and infections in compromised sockets.

**Figure 1:**
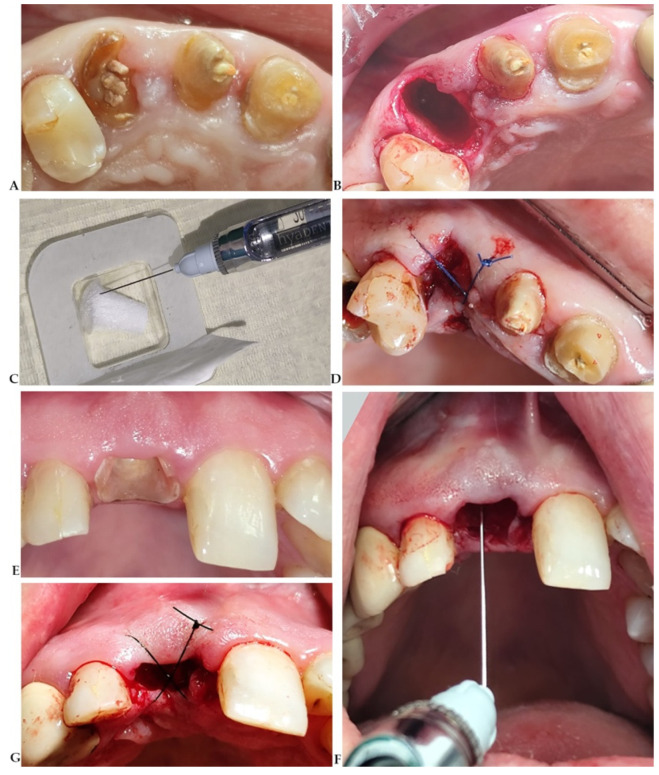
Alveolar ridge preservation in anterior zone. (**A**) Extensive destruction of the root in the coronal third of maxillary right canine; (**B**) post-extraction socket; (**C**) soaking the collagen sponge (Botiss biomaterials GmbH, Zossen, Germany) with HYA (hyaluronic acid) (Hyadent BC^®^, BioScience GmbH Dummer, Germany); (**D**) crossed suture over the preserved socket; (**E**) subgingival fracture of the maxillary right first incisor; (**F**) intra-socket HYA application; (**G**) HYA preserved socket.

Hyaluronan (also known as hyaluronic acid or hyaluronate, HYA) is a biological molecule recommended for preserving both fresh sockets and those affected by chronic pathologies [48]. Its osteoinductive, antimicrobial, and anti-inflammatory properties make it particularly beneficial for these purposes [49]. Additionally, HYA is often preferred over other biologics, such as bone morphogenetic protein-2, which has been associated with significant side effects [50]. HYA can be used alone or in combination with other biomaterials in ARP (Figure 1) to improve outcomes in post-extraction socket healing. However, the effects of HYA on ARP, especially regarding quantitative changes in soft tissue and bone, have been inconsistent across clinical and preclinical studies [48]. Therefore, HYA cannot be recommended preferentially over other materials for ARP. Given its notable biological effects, ease of clinical application, and potential for combination with other materials, an evaluation of recent data on HYA’s clinical impact on ridge preservation would be valuable for improving clinical practice.

The aim of this review is to assess the current clinical and preclinical evidence on the biological effects of HYA and evaluate its application in ARP, either alone or in combination with other products, with regard to its impact on hard and soft tissue dimensional changes, early wound healing, and implant success rates.

## 2. Materials and Methods

The review aimed to gather general information on the characteristics and biological effects of HYA in various oral therapeutic applications, based on the recent literature. Additionally, the review focused on evaluating the efficacy of HYA in ARP procedures, particularly in terms of the dimensional and qualitative benefits for bone and soft tissues, as reported in both clinical and preclinical studies. A comprehensive literature search was conducted to provide a detailed overview, as outlined below.

### 2.1. Information Sources and Literature Search

The narrative review was conducted through a comprehensive electronic literature search in two databases -Ovid and PubMed- with the last search performed up to 10 October 2024. The search focused on identifying current articles that examine the local application of HYA, both alone and in conjunction with additional biomaterials, in ARP techniques. A filter of publication dates for a period of 10 years was used for both searches with no further restrictions. The search strategy included the following MeSH terms, related keywords, and abbreviations combined with Boolean operators (“AND”, “OR”): “Hyaluronic Acid”, “Hyaluronan”, “Hyaluronate”, “Hyaluronate Sodium”, “Alveolar Ridge Augmentation”, “Alveolar Ridge Preservation”, “Ridge Preservation”, “Bone Augmentation”, “Bone Regeneration”, “Guided Tissue Regeneration”, “Alveolar Bone Grafting”, “Socket Preservation”, “Socket Seal”, “Socket Healing”, “Tooth Extraction”, “Tooth Socket”. The search in Ovid was performed using the same terms and Boolean operators. Additionally, the asterisk (*) served as a truncation symbol, while “adj” indicates the permissible number of words that can appear between keywords. The abbreviation “mp” encompasses several areas, for instance, title (“ti”), abstract (“ab”), or original title (“kf”).

### 2.2. Selection of the Studies

Two reviewers (ICM, CSC) independently screened the identified titles and abstracts to assess the eligibility of the studies based on the inclusion and exclusion criteria. If abstracts were unavailable or did not provide sufficient information to determine eligibility, but the titles indicated relevance to this study’s topic, the full texts were retrieved and evaluated for inclusion. The potentially relevant initially retrieved eligible articles were then compared by the reviewers, and any discrepancies were resolved through discussion with a third party, an experienced senior reviewer (AR), to ensure consensus. Articles with available full text were included in this narrative review.

Inclusion criteria refer to English-written articles published between 1 January 2014, and 10 October 2024, respecting the following framework:

Study type: human trials (prospective and retrospective studies, randomized clinical trials, case-control, and case series studies), systematic reviews of human trials, and preclinical studies.

Intervention: local HYA application in an ARP procedure of normal or altered sockets.

Outcomes: quantitative dimensional clinical, imagistic, or histomorphometric outcomes of the socket soft and hard tissues, histological qualitative data related to bone formation, and implant success rate.

The articles were excluded in the case of HYA applications other than ARP purposes, if only HYA was topically applied by patients and no ARP was conducted, or if only patient-centered outcomes related to ARP were reported.

### 2.3. Data Collection and Extraction

The articles’ content was evaluated after initial matching eligibility criteria. The two reviewers (ICM, CSC) thoroughly read the full texts and confirmed their eligibility for inclusion in this paper. Data were extracted and collected in a standardized spreadsheet (Microsoft Excel—Microsoft 365, Version 2401, Microsoft Corporation, Redmond, WA, USA). Any disagreements in the data extraction process were resolved through discussion with the third reviewer (AR) to ensure consistency. Information from the articles was extracted from the text, tables, and figures and grouped under the following categories:

(1) first author, (2) year of publication, (3) study design, (4) participants and their medical characteristics, (5) treatment groups and intervention details, (6) product information related to HYA and its pharmaceutical form, (7) outcomes (clinical, radiographical, histological, histomorphometric) in relation to the follow-up period.

## 3. Results

### 3.1. Hyaluronic Acid Molecule and Functions

HYA is a naturally occurring, high-molecular-weight glycosaminoglycan found in various body fluids, including synovial fluid, serum, saliva, and gingival crevicular fluid, as well as in the extracellular matrix of both mineralized and non-mineralized tissues, such as the skin, eyes, and periodontium. It is an essential structural component in both soft and hard oral tissues [51,52,53,54,55].

HYA has garnered interest in dentistry due to its biocompatibility, regenerative properties, antimicrobial and anti-inflammatory effects, and its ability to promote healing [55]. Among its physiological and structural functions, HYA plays crucial roles in extracellular and cellular interactions, interactions with growth factors, tissue lubrication, and regulation of osmotic pressure. It has a unique ability to bind with a variety of molecules, including proteins, lipids, and carbohydrates, thus contributing to the structural and homeostatic integrity of tissues [56]. As a hygroscopic substance, HYA can absorb and retain water molecules, enabling it to function as both a lubricant and a shock absorber, protecting tissues from mechanical stress [56].

HYA is involved in numerous tissue reactions, such as cell activation, neutralization, stimulation of cell proliferation, collagen synthesis, and inflammation. These effects, however, depend on the molecular size of HYA, its concentration, and the specific cell type targeted. Additionally, HYA plays a critical role as a scaffolding material, enhancing the biological properties of scaffolds used in bone regeneration [57]. The molecular weight of HYA is a key element in its effects on cells and tissues, influencing its properties and interactions. High molecular weight HYA is linked with homeostasis and protective action since low molecular weight HYA is related to a pathological condition in the tissue [58].

HYA modulates cell behavior by interacting with cell-associated hyaluronan-binding proteins, also known as hyaladherins, such as CD44. CD44 binding to HYA influences cell adhesion to extracellular matrix elements and stimulates aggregation, proliferation, migration, and angiogenesis [59,60].

HYA osteoinductive capacity has been previously reported [61]. HYA influences various signaling pathways that regulate osteogenesis and bone remodeling [62]. By interacting with cell surface receptors such as CD44 and Receptor for Hyaluronan Mediated Motility (RHAMM) [63], HYA can stimulate osteoblast activity and mineralization [64]. Additionally, HYA inhibits osteoclastogenesis and bone resorption, thereby preserving bone density [65]. The combination of HYA with the allogeneic bone substitutes might synergistically increase these osteogenic capabilities, resulting in higher bone density in the grafted area [66].

HYA has demonstrated antimicrobial [67] and anti-inflammatory [68] capacities. A bacteriostatic, but not bactericidal, effect of HYA on periodontal pathogens such as *Aggregatibacter actinomycetemcomitans*, *Porphyromonas gingivalis*, *Prevotella oris* [68], and on *Prevotella intermedia* [69] has been reported, *in vitro*. Another analysis did not demonstrate a bacteriostatic effect of HYA on *Porphyromonas gingivalis*. However, the bacteriostatic action of HYA appeared inferior to that of chlorhexidine [69]. In patients with periodontitis, the adjunctive use of HYA to subgingival mechanical instrumentation induced a persistent reduction of *Aggregatibacter actinomycetemcomitans* and *Campylobacter rectus*, after 6 months when compared to only mechanical instrumentation [70]. Opposite results were reported by others [71]. The HYA antibacterial effect could explain the reduction of inflammation after its use in gingivitis [72,73] or periodontitis [55,71] treatment. Reduction of inflammation may be also a direct interference with inflammation pathways since high molecular weight HYA within the tissues may exert an anti-inflammatory effect [55]. High molecular weight HYA inhibits mitogen-activated protein kinase (MAPK) and nuclear factor kappa-light-chain-enhancer of activated B cells (NF-κB) signaling pathways, implying anti-inflammatory properties through the production of IL-4 and IL-13. High molecular weight HYA also stimulates IL-10 production, thereby reducing inflammation in the tumor environment [58].

These properties could motivate clinicians in relation to HYA’s use in dentistry [56].

### 3.2. Common Applications of HYA in Dentistry

HYA induced favorable recovery and reduced morbidity after oral interventions like tooth extractions or implant placement surgeries. It also elevated bone density and osteogenesis in the maxillofacial region [56]. HYA has been reported to ameliorate osseointegration, implant stability, and longevity and diminish implant failure rates. It also minimizes inflammation during wound healing, promoting cell proliferation, reepithelialization, and scar reduction [56].

After endodontic surgeries, HYA can reduce postoperative discomfort and improve recovery. HYA has been applied in root canals in endodontics to favor the healing and regeneration of the periapical tissues following root canal treatment [56].

HYA ameliorated clinical outcomes when used in nonsurgical periodontitis therapy, such as reduction of pocket depth and gingival inflammation and improved attachment levels and ameliorated healing and tissue regeneration after periodontal surgeries [56]. Other data do not sustain the obtaining of clinical attachment gain after the adjunctive use of HYA to subgingival mechanical instrumentation [55]. Histological data provided by preclinical studies have demonstrated a positive effect on the healing of periodontal intrabony and gingival recession defects after HYA application [74,75].

HYA has been used in the treatment of oral ulcers in children in order to overcome discomfort. To assist in decreasing inflammation and improving healing, HYA can be used topically or as a mouthwash [56].

Injections of HA into the periodontal ligament have been found to promote osteoclast and osteoblast expression, encouraging faster tooth mobility during orthodontic treatment [56].

### 3.3. HYA Effects in Alveolar Ridge Preservation of Natural or Infected Sockets

#### 3.3.1. Study Selection and Characteristics

**Figure 2:**
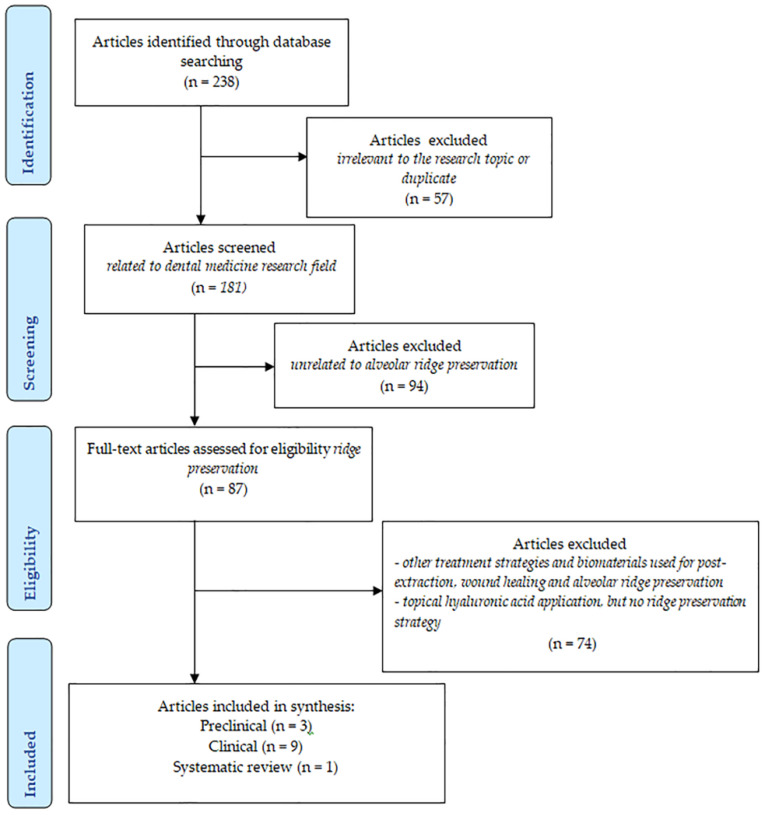
Flowchart of the included studies.

The electronic literature search in PubMed identified a total of 238 records. After excluding 57 articles unrelated to dentistry or duplicates, 181 publications remained for further screening based on titles, abstracts, or full texts when abstracts lacked sufficient detail to determine eligibility. This led to the exclusion of an additional 94 irrelevant studies, resulting in 87 articles on ARP biomaterials and techniques being reviewed in full to clarify eligibility. Within these 87 studies, 13 specifically examined the use of HYA—either alone or combined with other biomaterials—in ARP. This group consisted of 9 clinical trials, 3 preclinical studies, and 1 systematic review. Only clinical and preclinical studies were included in the present review and evaluated in detail (Figure 2). The included publications are available in Table 1 and Table 2.

Since the availability of the most recently published systematic review on this topic [48], three other clinical trials using HYA in ARP approaches have been published and included in the present review [66,82,83]. The publications analyzed by Domic et al. [48] that corresponded to the current inclusion criteria were also considered by the present narrative review.

#### 3.3.2. Study Intervention and HYA Information

**Figure 3:**
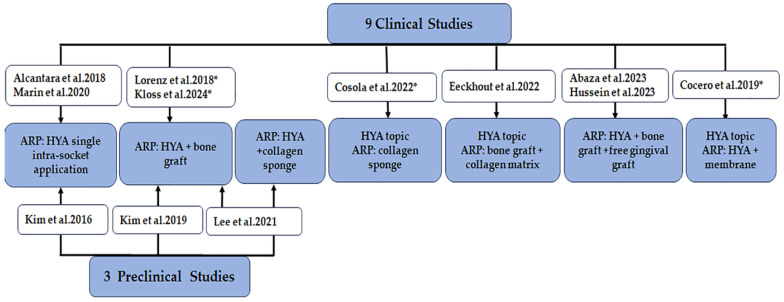
Distribution of studies depending on ARP intervention and HYA information (ARP, alveolar ridge preservation technique; HYA, hyaluronic acid-based product; *, unknown HYA-based product) [49,66,76,77,78,79,80,81,82,83,84,85].

The ARP approaches and the role of HYA in post-extraction interventions from the identified clinical and preclinical studies are briefly displayed in Figure 3.

Details on HYA pharmaceutical form and its concentration used by the identified studies, as well as its function in the ARP overall approach, are available in Table 1 and briefly synthesized below.

Five (*n* = 5) out of the nine clinical studies included in the present review used an HYA-based commercial product for which, in most of the cases, the pharmaceutical form was gel (*n* = 4) [76,79,80,82] with HYA concentrations ranging between 0.8% and 1.6%. From these five studies, in two studies (*n* = 2), the HYA-based gel was used as a single intra-socket biomaterial in ARP [76,79], in one study (*n* = 1), intra-socket HYA gel was used in combination with a commercial bone graft plus an external free gingival graft [82]; and in one study (*n* = 1), HYA gel was placed topically three times/day for 7 days onto the preserved sockets with a commercial collagen matrix plus a commercial xenograft [80]. One study (*n* = 1) from the five mentioning a commercial HYA-based product used a 2% HYA solution [83] that was mixed with a commercial bone graft and associated with an external free gingival graft for the ARP procedure (Table 1 and Figure 2).

Four clinical studies (*n* = 4) used self-made HYA products with unknown HYA concentrations [66,77,78,81]: 2 gel products [78,81] and 2 unknown pharmaceutical forms [66,77]. From these four studies, two (*n* = 2) of them applied HYA in the sockets in association with bone graft [66,77]; in one study (*n* = 1), HYA was placed intra-socket together with an external membrane and applied also topically three times a day for 7 days [78]; and in one study (*n* = 1), HYA was applied topically once a day for 15 days over the preserved sockets with a collagen sponge [81] (Table 1 and Figure 2).

All three preclinical studies (*n* = 3) used 1% HYA-based commercial gels as a single ARP approach [49] or in association with a commercial collagen sponge [84] or with a commercial bone substitute [85].

Only four (*n* = 4) out of nine clinical studies included in the present review quantified soft tissue healing and changes after ARP based on HYA [79,80,81,83].

#### 3.3.3. The Efficacy of HYA in Alveolar Ridge Preservation Procedures

The synthesis of the most relevant quantitative outcomes provided by the identified publications is available in Table 1 and is briefly explained below.

##### Clinical Bone- and Soft Tissue-Related Outcomes from Clinical Studies

In patients with liver failure, sockets treated with HYA and membrane showed significantly **smaller reductions in oral–buccal width** and **mesiodistal diameter** at 7, 14, and 21 days compared to natural healing (*p* < 0.001) [78].

ARP with HYA and xenograft resulted in significantly **less bone width loss** after four months (−0.56 ± 0.46 mm) compared to I-PRF and xenograft (−1.29 ± 0.58 mm) (*p* < 0.001) [83].

In patients with type 2 diabetes, HYA-treated sockets demonstrated significantly better **wound closure rates** than naturally healing sockets at 5 days (51.35 ± 18.35% vs. 29.11 ± 15.94%) (*p* < 0.001) and 25 days (84.36 ± 7.76% vs. 74.53 ± 12.94%) (*p* < 0.001) post-extraction [79]. HYA sockets also showed superior **wound healing scores** on day 15 (53.33% vs. 20%) (*p* = 0.021), while after 25 days, both groups exhibited high **healing rates** (76.67% vs. 63.33%) (*p* = 0.521) [79].

After ARP with collagen-enriched xenograft and collagen matrix sealing, with or without topical HYA, no significant improvements were observed in **wound-healing scores** (1.3 vs. 1.09) (*p* = 0.424) after 3 weeks, **buccal soft tissue height** (0.15 mm vs. 0.56 mm) (*p* = 0.226), or **oral soft tissue height** (0.28 mm vs. −0.14 mm) (*p* = 0.303) at 4 months. Additionally, **buccal soft tissue profile changes** were similar at four months (−1.13 mm vs. −1.06 mm) (*p* = 0.660) [80].

Two months after ARP with a type-I collagen sponge plus topical HYA, **soft tissue healing** was complete, with no significant differences in **soft tissue volume changes** compared to the collagen sponge group (95.85 ± 1.81% vs. 95.55 ± 1.88%) (*p* = 0.838). However, the collagen sponge with the HYA group showed significantly smaller edema and thus **soft tissue volume changes** at 7 days (105.05 ± 5.74% vs. 109.15 ± 6.3%) (*p* = 0.0380) [81].

After one year, preserved sites with HYA, xenograft, and free palatal graft exhibited a slight **decrease in soft tissue thickness** (−0.15 ± 0.08 mm), while the I-PRF with xenograft and palatal graft approach significantly increased thickness (0.21 ± 0.12 mm) (*p* < 0.001) [83].

##### Imagistic Bone-Related Outcomes from Clinical Studies

CBCT analysis showed significantly higher **newly formed bone** in HYA-preserved sockets compared to naturally healing sockets at 30 days (57.27% vs. 45.98%) (*p* = 0.004), but no differences at 90 days (85.83% vs. 83.25%) (*p* = 0.216). After 90 days, no significant differences in **bucco–lingual alveolar ridge width loss** were noted between HYA-preserved and naturally healing sockets across the cervical, middle, and apical thirds (*p* > 0.05) [76].

Another study showed that at 1 mm from the coronal margin, HYA plus bone graft plus matrix-treated sockets showed more **bone width shrinkage** than controls (3.55 mm vs. 1.92 mm) (*p* = 0.025) [80].

Four months post-treatment, HYA with xenograft and free palatal graft yielded significantly higher **bone width** (9.78 ± 0.87 mm) compared to I-PRF with xenograft (8.60 ± 1.27 mm) or xenograft alone (7.99 ± 0.89 mm) (*p* < 0.001) [83]. Additionally, these sockets exhibited a higher **area fraction of newly formed bone** (56.6 ± 7.35%) than I-PRFplus xenograft sites (28.74 ± 5.15%) or xenograft sites (24.05 ± 3.64%) (*p* < 0.001) [83].

No significant differences in radiographic **vertical bone loss** were observed at four months between collagen-enriched xenografts with collagen matrix sealing, with or without topical HYA care, at the buccal (1 mm vs. 0.45 mm) (*p* = 0.237) or oral (1.46 mm vs. 0.96 mm) (*p* = 0.351) sites [80].

However, CBCT revealed significantly lower **volumetric** and **linear bone resorption** (26.96 ± 1.83%; 0.73 ± 0.052 mm) in sockets preserved with HYA plus xenograft plus free palatal graft compared to xenograft with free palatal graft alone (36.56 ± 1.69%; 1.42 ± 0.16 mm) (*p* = 0.018) [82].

Recent data showed that HYA combined with xenograft and free palatal graft resulted in the **lowest crestal bone loss** (−0.33 ± 0.15 mm) compared to I-PRF with xenograft and free palatal graft (−0.53 ± 0.11 mm) and xenograft alone (−0.98 ± 0.18 mm) after 4 months (*p* < 0.001) [83]. CBCT scans further revealed significantly less **vertical bone height loss** in HYA with allogeneic bone graft and aPRF-treated sockets (−0.19 ± 0.51 mm) versus those with allogeneic bone graft and aPRF (−0.82 ± 0.95 mm) (*p* = 0.011) after 4 months. Additionally, the **graft shrinkage rate** was lower in the HYA with an allogeneic bone graft and a PRF group (10.3 ± 7.7%) compared to the allogeneic bone graft plus aPRF group (16.9 ± 11.5%) (*p* = 0.038), and radiographic bone density was significantly higher in the HYA plus allogeneic bone graft plus aPRF group (211.03 ± 67.35 Hounsfield Units (HU) vs. 132.66 ± 48.85 HU) (*p* = 0.004) [66].

##### Imagistic Bone-Related Outcomes from Preclinical Studies

Micro-CT analysis in rats showed significantly greater **ridge width at 1 mm** and **2 mm** from the coronal margin in the collagenated xenograft (16.92 ± 1.8 mm, 9.79 ± 6.57 mm) and HYA plus collagenated xenograft (15.91 ± 7.67 mm, 6.15 ± 3.72 mm) groups compared to the collagen sponge and HYA plus collagen sponge groups after one month (*p* = 0.001). After three months, **ridge width at 1 mm** and **2 mm** remained significantly higher in the HYA plus collagenated xenograft group (9.39 ± 9.27 mm, 8.98 ± 7.25 mm) compared to collagen sponge groups (*p* = 0.001 and *p* = 0.012, respectively) [85].

##### Implant-Related Performance

Implant placement in sockets preserved with an injectable bone substitute, HYA, and collagen matrix showed a 100% **survival rate** and minimal **marginal bone loss** (0.136 mm) after one year of prosthetic loading [77].

No significant differences in **implant success** [86] were observed between the HYA plus allogeneic bone graft with aPRF and allogeneic bone graft with aPRF groups. **Radiographic bone loss** was <2 mm in most implants for both groups after one year (*p* = 0.523) [66].

##### Histomorphometric Data from Clinical Studies

In humans, ARP with injectable bone graft and HYA resulted in high amount of **newly formed bone** (44.92% ± 5.16%) with minimal **residual graft material** (2.59% ± 2.05%) (*p* < 0.01) [77]. Similarly, HYA plus xenograft plus free palatal graft preserved sockets showed significantly more **newly formed bone** (56.6% ± 7.35%) and less **residual graft material** (2.6 ± 1.27%) compared to I-PRF plus xenograft plus free palatal graft alveolae (28.74 ± 5.15% and 6.76 ± 2.59%) or xenograft alone (24.05 ± 3.64% and 2.71 ± 1.24%) (*p* < 0.001) [83].

##### Histomorphometric Data from Pre-Clinical Studies

Ridge preservation in dog sockets with chronic pathology showed significantly higher **mineralized bone** in HYA sockets (63.29 ± 9.78%) compared to natural healing (47.80 ± 6.60%) afterthree months (*p* < 0.05) [49].

**Micro-CT** analysis revealed alveolar bone overgrowth in HYA plus collagen sponge and rh-BMP-2 plus collagen sponge sockets (11.73 ± 4.73%, 15.94 ± 3.12%), while naturally healing and collagen sponge sockets showed bone loss (−10.74 ± 1.78%, −6.55 ± 9.82%) [84]. No significant differences were found between HYA plus collagen sponge and rh-BMP-2 + collagen sponge sockets (*p* < 0.05). At three months, **bone volume density** values showed no significant differences among HYA plus collagen sponge (20.06 ± 6.27%), rh-BMP-2 plus collagen sponge (20.11 ± 6.64%), naturally healing (18.00 ± 6.62%), and collagen sponge-only sockets (17.89 ± 6.02%) (*p* > 0.05) [84]. 

After one month, in socket preservation in dogs with four different approaches, HYA plus collagen sponge sockets had the highest **mineralized bone** proportion (62.97 ± 4.39%) (*p* < 0.05) compared to the other treatment groups and significantly greater **new bone formation** and collagen sponge group (17.73 ± 10.36% vs. 7.14 ± 1.84%) (*p* = 0.043) [85]. After three months, ARP in rats showed HYA plus collagen sponge sockets had more **mineralized bone** than collagen sponge (64.69% ± 3.98% vs. 45.19% ± 3.06%) and collagenated xenograft sockets (41.89% ± 5.03%) (*p* = 0.001), but not HYA plus collagenated xenograft sockets (59.93% ± 5.44%) (*p* = 0.405). The **newly formed bone** was higher in HYA plus collagen sponge vs. collagen sponge (15.53% ± 2.41% vs. 7.53% ± 2.19%) (*p* < 0.05) and in HYA plus collagenated xenograft vs. xenograft alone (11.30% ± 3.06% vs. 5.57% ± 1.44%) (*p* = 0.021). **Residual graft particles** were similar between xenograft groups (*p* = 0.53) [85].

#### 3.3.4. Histological Data Associated with HYA Application in Post-Extraction Sockets

Description of histological samples of preserved sockets superposed with the data from the histomorphometric analysis provided in the section above. Some details should be added.

In HYA-preserved compromised sockets of dogs, more osteoblasts in the periphery of the mineralized bone were observed compared to natural healing sockets, and osteoclasts were highlighted in the HYA-preserved sockets [49].

In the HYA plus collagen sponge and rh BMP-2 plus collagen sponge sockets, a higher density of mineralized bone than in natural healing or absorbable collagen sponge sockets was observed, with a continuous coronal mineralized line of new and old cortical bone. Additionally, in these groups of sockets, more osteoblasts were seen at the periphery of the newly formed bone, and more osteocalcin-positive cells, mostly at the periphery of the bone trabeculae, were present; osteoclasts were seen in their lacunae [84]. A cortical cap at the entrance of the sockets was also observed for sockets preserved with HYA plus collagen sponge, HYA plus collagenated xenograft, collagen sponge, and collagenated xenograft [85]. A highly interconnected trabecular pattern was observed in the HYA plus collagen sponge and HYA plus collagenated xenograft groups [85].

On the contrary, in the naturally healing and absorbable collagen sponge groups, the cortication of bone covering the alveolar crest was not prominent or mature. The line of cortication was not continuous with adjacent cortical bone. Most of the mineralized tissue was fine trabeculae, which consisted of primary osteons and lamellar bone. In the periphery of the newly formed bone, the number of osteoblasts was low [84].

## 4. Discussion

A recent systematic review and meta-analysis revealed that although preclinical studies demonstrated promising outcomes for HYA as an adjunct in tooth extraction, these findings have not been consistently mirrored in clinical trials regarding its effects on reducing alveolar ridge remodeling or promoting bone regeneration [48]. However, the meta-analysis highlighted certain positive effects of HYA on patient-centered outcomes and improved soft tissue healing following ARP [48]. Despite these findings, the potential of HYA to enhance wound healing, attributed to its anti-bacterial, anti-inflammatory, and bone-forming properties, continues to draw interest from both clinicians and researchers. This underlines the importance of further evaluations of HYA in the context of ARP, as explored in the present review.

The clinical and preclinical studies included in the present review reported significantly improved newly formed bone after HYA intra-socket application as a single approach after 30 days (*p* **=** 0.004) [76] as well as at four months after HYA associated with a bone graft plus free palatal graft (*p* < 0.01) [83] and at three months after ARP with HYA plus collagen sponge or HYA plus bone graft (p = 0.008) [85]. As compared with controls, significantly more mineralized bone formed three months after ARP with HYA (*p* < 0.05) [49], HYA plus collagen sponge, or HYA plus bone graft (*p* = 0.001) [85]. As compared with controls, ARP with HYA plus a bone graft plus a free palatal graft was associated with significantly more mature bone, less clinical bone width changes at one year, and less radiographical crestal bone loss at 4 months (*p* < 0.01) [83] as well as less radiological volumetric and linear bone resorption after 4 months (*p* = 0.018) [82]. When HYA was associated with a bone graft plus aPRF, significantly less bone height loss (*p* **=** 0.011) and improved bone density (*p* = 0.004) were highlighted after four months as compared with controls [66].

Only one pre-clinical study, Kim et al. (2016) [49], and two clinical studies, Alcantara et al. (2018) and Marin et al. 2020 [76,79], investigated the effect of HYA as a single product in ARP. Although the current data on the benefits of HYA in ARP as a standalone treatment is limited, its significant healing properties and complex biological interactions suggest that further investigation in this area is warranted. Given its ease of use in clinical settings and its demonstrated efficacy in promoting bone formation, the application of HYA in ARP could be considered a viable option for clinical practice.

When used alongside other biomaterials, it is inherently challenging to measure the impact of HYA, particularly since studies indicate that xenografts and allografts, when combined with a collagen membrane or sponge, tend to achieve the most positive results in ARP [34]. Often used in combination with ARP, bone graft materials provide a scaffold for the new bone formation, while the membrane guides the tissue regeneration [37]. Moreover, the application of a-PRF, the last generation of the solid form of PRF, used as an adjunct to ARP, was shown to have a positive impact on increasing bone density. However, the exact role of PRF is still not clear [40]. Therefore, according to the findings of the studies included, it is possible that HYA might not have a significant impact when used alone, especially in the long term. However, in combination with other biomaterials, it might have the potential to be an effective adjunct in ARP.

HYA is associated with a membrane-induced significant reduction in clinical mesio-distal and oral–buccal ridge diameter in short-term follow-ups (*p* < 0.001) [78]. A protective effect on ridge dimensional changes seems to be induced by HYA as demonstrated by short-term and long-term follow-up reports. This may be due to HYA’s ability to enhance tissue regeneration and wound healing [87]. The sequence of biologic events that developed during socket healing and remodeling is conducted by an interplay of various cytokines, chemokines, and growth factors directing cellular recruitment via the activation of signaling pathways characteristic for intramembranous osseous healing in which HYA induces a well-established influence [8,88].

Data from the studies included in the present review suggest that HYA may limit post-extraction alveolar bone resorption when mixed with bone graft material [66,82]. This is because the incorporation of HYA in bone grafts for ARP might facilitate better integration of the graft material with the surrounding tissues, leading to improved stability and outcomes [66]. It, therefore, seems that HYA might be an essential material in optimizing bone healing and preservation, which may further improve the predictability and long-term success of implant placement.

On the contrary, other reports showed no significant difference in radiographic bucco–lingual ridge width after HYA intra-socket application as a single approach versus naturally healing sockets after three months [76]. Neither topically applied HYA over the preserved alveoli with bone graft and collagen matrix led to significant radiographic improvements of the vertical crestal dimensional changes (*p* = 0.237) or horizontal bone shrinkage (*p* = 0.025) in favor of control sites after four months [80], possibly because the only difference between test and control sockets was HYA topical applications.

The results on HYA influence on soft tissues after ARP are more heterogeneous. In patients with type 2 diabetes, HYA intra-socket application as a single ARP approach induced significantly better wound healing and wound closure after 15 days (*p* **=** 0.021, *p* < 0.01) as compared with natural healing sockets [79]. The good short-term healing outcomes of the sockets treated with HYA indicate its significant role in improving post-extraction wound healing in the first two weeks post-surgery, which recommends HYA be used in ARP approaches in patients with diabetes. This time frame is the moment in which the patient with diabetes has a greater risk of superinfection [25,89], and, therefore, it is fundamental to have a fast and predictable wound-healing process.

HYA associated with a bone graft and free palatal graft did not influence the soft tissue thickness after four months (*p* = 0.516) and one year (*p* = 0.621) but induced significantly reduced differences in soft tissue thickness as compared with controls (*p* < 0.001) [83]. It would have been expected that the association of the free graft in the context of minimally invasive ARP approaches would enhance the quality and the quantity of the soft tissues as the literature reported [6,90].

HYA topical applications onto preserved sockets with collagen sponge versus collagen sponge sockets [81] or HYA topical applications after bone grafting plus collagen matrix versus bone grafting plus collagen matrix [80] did not induce significant benefits in terms of soft tissue volumetric changes after two months (*p* = 0.838) [81] or on wound healing after one and three weeks (*p* = 0.737, *p* = 0.424) [80].

Inconsistent outcomes related to soft tissue changes after HYA use by different ARP approaches reported by the present review are summed up with other data reported so far related to other ARP procedures [33,47,91]. This lack of difference has been attributed to a greater extent to soft tissue thickening in spontaneous healing sites, especially when the buccal osseous wall is thinner than 1 mm [19,47,91].

As such, from a clinical decision-making point of view, the choice to use HYA either alone or in combination with other biomaterials in ARP should be based on a comprehensive evaluation of the specific patient’s profile. Various factors, such as interproximal bone height, the thickness and integrity of the buccal bone plate, the condition of the remaining walls of the extraction socket, the contour of the soft tissue in relation to the underlying bone, and the gingival phenotype, should be considered when choosing an ARP approach [37]. The clinician’s judgment is essential in selecting the most suitable ARP technique to minimize bone loss following tooth extraction, preserve alveolar ridge dimension, and ensure successful implant placement and long-term implant performance [20].

The present narrative review has certain limitations stemming from variations in HYA concentrations, pharmaceutical formulations, application methods, evaluation techniques, surgical approaches, investigated patients’ characteristics, and short-term follow-up periods across the included studies. These discrepancies likely contributed to the heterogeneity of the results, making it difficult to draw definitive clinical conclusions. Another limitation is that the review included studies addressing both intact alveoli and those affected by pathological conditions, introducing further variability. Additionally, the general health status of the patients varied across studies, which undoubtedly influenced individual healing patterns and outcomes. The reliance on only two databases may have restricted the number of available studies. To better assess the effectiveness of HYA in promoting soft tissue healing and minimizing bone dimensional changes after ARP, further long-term follow-up clinical studies and preclinical studies with more precise, standardized, and transparent methodologies are needed.

## 5. Conclusions

Adding HYA to bone grafts could enhance the outcomes of ridge preservation. HYA could counteract the delayed healing resulting from grafting the sockets with bone substitutes, eventually reducing the time till implant surgery.

HYA seems promising in improving the quality of early wound healing after tooth extraction in patients with diabetes.

ARP with HYA plus absorbable collagen sponge may enhance the regenerative efficacy of bone and may be expected to change the adverse bone configuration of compromised infected sockets.

Some data reported that HYA could induce more important improvements in clinical and radiographic bone maturation-related outcomes in post-extraction sockets as compared with platelet-rich fibrin.

## Figures and Tables

**Table 1:** Clinical, radiographic, and histometric outcomes after the use of hyaluronic acid in extraction socket treatment, from clinical studies.

Study and Design	Ridge Preservation Method Treatment Groups HYA Application form Commercial Product	Investigation Type Outcome Parameters Follow-Up Moments	Outcomes				
Alcantara et al. 2018 [76] RCT, split-mouth, Humans	Test group: ARP with 1% HYA gel (Nikkol, BSPharma, Belo Horizonte, Brazil) Control group: Natural healing	Radiographic assessment (CBCT scan) of alveolar dimensional changes, percentage of newly formed bone and fractal dimension Day 30, 90		Test group	Control group		
			Newly formed bone (%), 30 d	57.27	45.98	*p* = 0.004	
			Newly formed bone (%), 90 d	85.83	83.25	*p* = 0.216	
			Buccolingual alveolar ridge width at the cervical/middle/apical thirds (mm), 90 d	0.71/0.25/0.19	0.75/0.54/0.21	*p* > 0.005	
			Fractal dimension, 30 d	1.098 ± 0.042	1.074 ± 0.045	*p* = 0.003	
			Fractal dimension, 90 d	No significant	differences	*p* > 0.005	
Lorenz et al. 2018 [77] Clinical trial, Humans	Treatment group: ARP with bone substitute (β-TCP + Methylcellulose) + HYA (Unknown commercial product) + collagen matrix (Unknown commercial product)	Clinical assessment of implant survival/success Radiographic assessment of implant marginal bone loss Histology/morphometry 4 months for ARP 1 year for implant success	Implant survival rate, 1 y		100%		
			Marginal bone loss (mm), 1 y		0.136		
			Amount of newly formed bone tissue (%), 4 m		44.92 ± 5.16		
			Amount of connective tissue (%), 4 m		52.49 ± 6.43		
			Amount of residual graft biomaterial (%), 4 m		2.59 ± 2.05		
Cocero et al. 2019 [78] RCT, split-mouth Humans	Test group: ARP with HYA gel (HYA, amino acids L-lysine, L-proline, L-leucine, glycine) (Unknown commercial product) + membrane (Unknown commercial product) + topical HYA gel Control group: Natural healing *Patients with liver failure*	Clinical assessment of the reduction of alveolar dimensions Day 7, 14, 21		Test group	Control group		
			Oral-buccal diameters (mm), 7 d	3.89 ± 1.73	4.64 ± 2.03	*p* < 0.0001	
			Oral-buccal diameters (mm), 14 d	2.09 ± 1.31	3.07 ± 1.51	*p* < 0.0001	
			Oral-buccal diameters (mm), 21 d	0.58 ± 1.11	1.21 ± 1.25	*p* < 0.0001	
			Mesio-distal diameters (mm), 7 d	3.61 ± 1.84	4.69 ± 2.04	*p* < 0.0001	
			Mesio-distal diameters (mm), 14 d	1.74 ± 1.54	2.82 ± 1.7	*p* < 0.0001	
			Mesio-distal diameters (mm), 21 d	0.44 ± 1.02	1.16 ± 1.25	*p* < 0.0001	
Marin et al. 2020 [79] RCT, split-mouth Humans	Test group: ARP with 0.8% HYA (Gengigel Forte®, Ricerfarma srl, Milano, Italy) Control group: Natural healing *Patients with poorly controlled type 2 diabetes*	Clinical assessment of wound closure rate and wound healing scale Day 5, 10, 15, 20, 25		Test group	Control group		
			Wound closure rate (%), 5 d	51.35 ± 18.35	29.11 ± 15.94	*p* < 0.001	
			Wound closure rate (%), 15 d	74.86 ± 11.31	61.61 ± 15.78	*p* < 0.001	
			Wound closure rate (%), 25 d	84.36 ± 7.76	74.53 ± 12.94	*p* < 0.001	
			Wound healing scale (%), 5 d (excellent)	16.67	3.33	*p* = 0.069	
			Wound healing scale (%), 15 d (excellent)	53.33	20	*p* = 0.021	
			Wound healing scale (%), 25 d (excellent)	76.67	63.33	*p* = 0.521	
Eeckhout et al. 2022 [80] RCT Humans	Test group: ARP with xenograft (Bio-Oss Collagen®, Geistlich Pharma AG, Wolhusen, Switzerland) + collagen matrix (Mucograft Seal®, Geistlich Pharma AG) + topical 0.8% HYA (Gengigel Forte®, Ricerfarma srl) Control group: ARP with xenograft (Bio-Oss Collagen®, Geistlich-Pharma AG) + collagen matrix (Mucograft Seal®, Geistlich-Pharma AG)	Clinical assessment of wound healing (examination and intra-oral scan) Radiographic assessment (CBCT scan) of bone dimensions 1 week 3 weeks 4 months		Test group	Control group		
			Bucco-lingual wound reduction (mm), 1 w	4.26	3.63	*p* > 0.005	
			Bucco-lingual wound reduction (mm), 3 w	0.77	1.03	*p* = 0.259	
			Mesio-distal wound reduction (mm), 1 w	2.00	2.2	*p* > 0.005	
			Mesio-distal wound reduction (mm), 3 w	0.57	0.49	*p* = 0.259	
			Ridge width, 1 mm coronal, (mm), 4 m	3.57	6.74	*p* = 0.025	
			Ridge width, 3 mm coronal, (mm), 4 m	6.37	8.36	*p* = 0.016	
			Ridge width, 5 mm coronal, (mm), 4 m	8.13	9.01	*p* = 0.213	
			Horizontal bone shrinkage, (mm), 4 m	3.55	1.92	*p* = 0.025	
			Buccal bone height shrinkage (mm), 4 m	1	0.45	*p* = 0.237	
			Oral bone height shrinkage (mm), 4 m	1.46	0.96	*p* = 0.351	
			Buccal soft tissue height (mm), 4 m	1.99	2.71	*p* = 0.226	
			Oral soft tissue height (mm), 4 m	2.38	1.62	*p* = 0.303	
			Soft tissue profile changes (mm), 4 m	−1.13	−1.06	*p* = 0.660	
			Buccal soft tissue height changes, 4 m	0.15	0.56	*p* = 0.226	
			Oral soft tissue height changes (mm), 4 m	0.28	−0.14	*p* = 0.303	
			Socket wound healing score, 1 w	1.87	1.96	*p* = 0.737	
			Socket wound healing score, 3 w	1.3	1.09	*p* = 0.424	
Cosola et al. 2022 [81] RCT, Humans	Test group: Collagen sponge (Condress®, Smith & Nephew Srl Monza, Italy) + topical HYA gel (HYA aminoacid, -Polifarma Benesser & Professional Dietetics, Italy) Control group: Collagen sponge (Condress®)	Clinical assessment of swelling and soft tissue healing rate through 3D intra-oral scanner Day 7, 14 1 month 2 months		Test group	Control group		
			Volume change of the soft tissue (%). 7 d	105.05 ± 5.74	109.15 ± 6.3	*p* = 0.038	
			Volume change of the soft tissue (%), 2 m	95.85 ± 1.81	95.55 ± 1.88	*p* = 0.838	
Husseini et al. 2023 [82] RCT, Humans	Test group: ARP with xenograft (Bio-Oss®, Geistlich-Pharma AG) + 1.6% HYA gel (Hyadent BG, Regedent AG, Zurich, Switzerland) + free palatal graft Control group: ARP - xenograft (Bio-Oss®) + free palatal graft	Radiographic assessment (CBCT scan) of volumetric and linear bone resorption Histologic assessment of the newly formed bone and residual graft biomaterial 4 months		Test group	Control group		
			Volumetric bone resorption value (%), 4 m	26.96 ± 1.83	36.56 ± 1.69	*p* = 0.018	
			Linear bone resorption value (mm) (%), 4 m	0.73 ± 0.052	1.42 ± 0.16	*p* = 0.018	
Abaza et al. 2023 [83] RCT, Humans	Test group 1: ARP - Xenograft (Cerabone®, Botiss biomaterials GmbH, Zossen, Germany) + Injectable Platelet-Rich Fibrin+ Free palatal graft Test group 2: ARP -Xenograft (Cerabone®) + HYA gel solution (Perfectha, Sinclair Pharma, Chester, UK) + Free palatal graft Control group: ARP with Xenograft (Cerabone®) + Palatal free graft	Radiographic assessment (CBCT scan) of bone width and crestal bone height Clinical assessment of horizontal bone width and soft tissue thickness Histologic and morphometric assessment of the newly formed bone and residual graft biomaterial 4 months, 1 year		Test group 1	Test group 2	Control group	
			Radiographic bone width (mm), 4 m	8.60 ± 1.27	9.78 ± 0.87	7.99 ± 0.89	*p* = 0.007
			Radiographic crestal bone loss (mm), 4 m	−0.53 ± 0.11	−0.33 ± 0.15	−0.98 ± 0.18	*p* < 0.001
			Clinical bone width (mm), 4 m	6.38 ± 1.16	6.94 ± 1.18	6.00 ± 1.81	*p* = 0.42
			Clinical bone width (mm), 1 y	6.27 ± 0.36	6.88 ± 1	6.00 ± 0.9	*p* = 0.700
			Difference of clinical bone width (mm), 1 y	−1.29 ± 0.58	−0.56 ± 0.46	−0.44 ± 1.35	*p* < 0.001
			Clinical soft tissue thickness (mm), 4 m	1.62 ± 0.44	1.50 ± 0.46	1.75 ± 0.38	*p* = 0.516
			Clinical soft tissue thickness (mm), 1 y	1.59 ± 0.33	1.47 ± 0.50	1.66 ± 0.31	*p* = 0.621
			Difference soft tissue thickness (mm), 1 y	0.21 ± 0.12	−0.15 ± 0.08	−0.9 ± 0.00	*p* < 0.001
			Mean area fraction newly formed bone (%), 4 m	28.74 ± 5.15	56.6 ± 7.35	24.05 ± 3.64	*p* < 0.001
			Mean area fraction of mature bone (%), 4 m	7.51 ± 3.63	18.26 ± 4.44	2.41 ± 1.36	*p* < 0.001
			Mean area fraction of residual graft (%), 4 m	6.76 ± 2.59	2.63 ± 1.27	2.71 ± 1.24	*p* < 0.001
Kloss et al. 2024 [66] Clinical trial Humans	Test group: ARP with allograft (Granular maxgraft®, Botiss biomaterials GmbH) + HYA (Unknown commercial product) + Advanced Platelet Rich Fibrin Control group: ARP with allograft (Granular maxgraft®) + Advanced Platelet Rich Fibrin	Radiographic assessment (CBCT scan) of vertical and horizontal bone loss, graft stability, graft shrinkage rate and bone density Clinical and radiographic assessment of implant survival/success 4 months for alveolar ridge preservation 1 year for implant survival and success		Test group	Control group		
			Bone height loss (mm), 4 m	−0.19 ± 0.51	−0.82 ± 0.95	*p* = 0.011	
			Graft shrinkage rate (%), 4 m	10.3 ± 7.7	16.9 ± 11.5	*p* = 0.038	
			Bone density (HU), 4 m	211.03 ± 67.35	132.66 ± 48.85	*p* = 0.004	
			Implant quality scale (success), 1 y	21 implants out of 21	18 implants out of 19	*p* = 0.475	
			Radiographic periimplant bone loss < 2 mm, 1 y	13 implants out of 21	10 implants out of 19	*p* = 0.523	

Abbreviations: ARP = alveolar ridge preservation; β-TCP = β-Tricalcium phosphate; d = day; HYA = Hyaluronic acid; HU = Hounsfield Units; m = month; mm = millimeters; % = percentage; RCT = randomized clinical trial; y = year.

**Table 2:** Clinical, radiographic, and histometric outcomes after the use of hyaluronic acid in extraction socket treatment, from preclinical studies.

Study and Design	Ridge Preservation Method Treatment Groups HYA Application form and Commercial Product Information	Investigation Type Outcome Parameters Follow-Up Moments	Outcomes					
Kim JJ et al. 2016 [49] Preclinical study Dogs	Test group: Preservation with 1% HYA gel (Healon, Pharmacia & Upjohn, Upsala, Sweden) Control group: Natural healing *Infected sockets*	Clinical assessment of wound healing. Histological/morphometric assessment 3 months		Test group	Control group			
			Mineralized bone (%), 3 m	63.29 ± 9.78	47.80 ± 6.60	*p* < 0.05		
			Bone marrow (%), 3 m	34.73 ± 8.97	50.4 ± 6.38	*p* < 0.057		
Kim JJ et al. 2019 [84] Preclinical study Dogs	Test group 1: Collagen sponge (Teruplug®, Olympus Terumo Biomaterials Corporation, Tokyo, Japan) Test group 2: Collagen sponge (Teruplug®) + 1%HYA gel (Healon) Test group 3: Collagen sponge (Teruplug®) + recombinant human bone morphogenetic protein-2 (O-BMP®, Osstem Implant Co., Busan, Korea) Control group: Natural healing *Infected sockets*	Radiographic (Micro-CT)/morphometric assessment Histomorphometry Immunohistochemical assessment of bone formation 3 months		Test group 1	Test group 2	Test group 3	Control group	
			Net Area (%), 3 m	−6.55 ± 9.82	11.73 ± 4.73	15.94 ± 3.12	−10.74 ± 1.78	*p* < 0.05
			Bone volume density (%), 3 m	17.89 ± 6.02	20.06 ± 6.27	20.11 ± 6.64	18.00 ± 6.62	*p* > 0.05
			Immune positive cells for osteocalcin (n), 3 m	83.00 ± 27.56	319.00 ± 138.63	281.67 ± 125.74	88.67 ± 43.00	*p* < 0.05
Lee JB et al. 2021 [85] Preclinical study Rats	Test group 1: Collagen sponge (Teruplug®) Test group 2: Collagen sponge (Teruplug®) + 1% HYA gel (Healon) Test group 3: Deproteinized bovine bone mineral, 10% collagen (Bio-Oss Collagen®, Geistlich-Pharma Wolhusen, Switzerland) Test group 4: Deproteinized bovine bone mineral with 10% collagen (Bio-Oss Collagen®) + 1% HYA gel (Healon)	Histologic assesement of mineralized bone formation Morphometric assessment of mineralized bone, newly formed bone, connective tissue, residual graft particles. Radiographic (Micro-CT)/morphometric assessment 1 months 2 months		Test group 1	Test group 2	Test group 3	Test group 4	
			Mineralized bone (%), 1 m	34.61 ± 13.0	62.97 ± 4.39	43.58 ± 6.65	46.10 ± 9.73	*p* = 0.024
			Mineralized bone (%), 3 m	45.19 ± 3.06	64.69 ± 3.98	41.89 ± 5.03	59.94 ± 5.44	*p* = 0.002
			Newly bone form (%), 1 m	7.14 ± 1.84	17.73 ± 10.36	5.93 ± 2.46	16.82 ± 6.84	*p* = 0.033
			Newly bone form (%), 3 m	7.53 ± 2.19	15.53 ± 2.41	5.57 ± 1.44	11.30 ± 3.06	*p* = 0.008
			Connective tissue (%), 1 m	61.15 ± 24.36	33.18 ± 27.00	44.34 ± 9.22	33.08 ± 13.98	*p* = 0.145
			Connective tissue (%), 3 m	17.07 ± 6.79	10.82 ± 4.96	35.05 ± 10.49	12.26 ± 5.55	*p* = 0.002
			Residual graft particles (%), 1 m	-	-	2.75 ± 1.35	1.78 ± 0.78	*p* = 0.225
			Residual graft particles (%), 3 m	-	-	3.71 ± 1.39	2.96 ± 2.03	*p* = 0.456

Abbreviations: HYA = hyaluronic acid; m = month; % = percentage.

## Data Availability

Not applicable.

## References

[1] Araujo M.G., Lindhe J. (2005). Dimensional ridge alterations following tooth extraction. An experimental study in the dog. J. Clin. Periodontol..

[2] Tan W.L., Wong T.L., Wong M.C., Lang N.P. (2012). A systematic review of post-extractional alveolar hard and soft tissue dimensional changes in humans. Clin. Oral Implants Res..

[3] Kim J.J., Ben Amara H., Chung I., Koo K.T. (2021). Compromised extraction sockets: A new classification and prevalence involving both soft and hard tissue loss. J. Periodontal Implant Sci..

[4] Van der Weijden F., Dell’Acqua F., Slot D.E. (2009). Alveolar bone dimensional changes of post-extraction sockets in humans: A systematic review. J. Clin. Periodontol..

[5] Trombelli L., Farina R., Marzola A., Bozzi L., Liljenberg B., Lindhe J. (2008). Modeling and remodeling of human extraction sockets. J. Clin. Periodontol..

[6] Jung R.E., Ioannidis A., Hämmerle C.H.F., Thoma D.S. (2018). Alveolar ridge preservation in the esthetic zone. Periodontol. 2000.

[7] Schropp L., Wenzel A., Kostopoulos L., Karring T. (2003). Bone healing and soft tissue contour changes following single-tooth extraction: A clinical and radiographic 12-month prospective study. Int. J. Periodontics Restor. Dent..

[8] Mardas N., Macbeth N., Donos N., Jung R.E., Zuercher A.N. (2023). Is alveolar ridge preservation an overtreatment?. Periodontol. 2000.

[9] Couso-Queiruga E., Stuhr S., Tattan M., Chambrone L., Avila-Ortiz G. (2021). Post-extraction dimensional changes: A systematic review and meta-analysis. J. Clin. Periodontol..

[10] Fok M.R., Jin L. (2024). Learn, unlearn, and relearn post-extraction alveolar socket healing: Evolving knowledge and practices. J. Dent..

[11] Morjaria K.R., Wilson R., Palmer R.M. (2014). Bone healing after tooth extraction with or without an intervention: A systematic review of randomized controlled trials. Clin. Implant Dent. Relat. Res..

[12] Barootchi S., Wang H.-L., Ravida A., Ben Amor F., Riccitiello F., Rengo C., Paz A., Laino L., Marenzi G., Gasparro R. (2019). Ridge preservation techniques to avoid invasive bone reconstruction: A systematic review and meta-analysis. Int. J. Oral Implantol..

[13] Barootchi S., Tavelli L., Majzoub J., Stefanini M., Wang H.L., Avila-Ortiz G. (2023). Alveolar ridge preservation: Complications and cost-effectiveness. Periodontol. 2000.

[14] Horvath A., Mardas N., Mezzomo L.A., Needleman I.G., Donos N. (2013). Alveolar ridge preservation. A systematic review. Clin. Oral Investig..

[15] Jambhekar S., Kernen F., Bidra A.S. (2015). Clinical and histologic outcomes of socket grafting after flapless tooth extraction: A systematic review of randomized controlled clinical trials. J. Prosthet. Dent..

[16] Ten Heggeler J.M., Slot D.E., Van der Weijden G.A. (2011). Effect of socket preservation therapies following tooth extraction in non-molar regions in humans: A systematic review. Clin. Oral Implant Res..

[17] Natto Z.S., Parashis A., Steffensen B., Ganguly R., Finkelman M.D., Jeong Y.N. (2017). Efficacy of collagen matrix seal and collagen sponge on ridge preservation in combination with bone allograft: A randomized controlled clinical trial. J. Clin. Periodontol..

[18] Chatzopoulos G.S., Koidou V.P., Sonnenberger M., Johnson D., Chu H., Wolff L.F. (2024). Postextraction ridge preservation by using dense PTFE membranes: A systematic review and meta-analysis. J. Prosthet. Dent..

[19] Chappuis V., Araujo M.G., Buser D. (2017). Clinical relevance of dimensional bone and soft tissue alterations post-extraction in esthetic sites. Periodontol. 2000.

[20] Tonetti M.S., Jung R.E., Avila-Ortiz G., Blanco J., Cosyn J., Fickl S., Figuero E., Goldstein M., Graziani F., Madianos P. (2019). Management of the extraction socket and timing of implant placement: Consensus report and clinical recommendations of group 3 of the XV European Workshop in Periodontology. J. Clin. Periodontol..

[21] Kingsmill V. (1999). Post-extraction remodeling of the adult mandible. Crit. Rev. Oral Biol. Med..

[22] Kim T., Kim S., Song M., Lee C., Yagita H., Williams D.W., Sung E.C., Hong C., Shin K.-H., Kang M.K. (2018). Removal of pre-existing periodontal inflammatory condition before tooth extraction ameliorates medication-related osteonecrosis of the jaw-like lesion in mice. Am. J. Pathol..

[23] Tsigarida A., Toscano J., Bezerra B.d.B., Geminiani A., Barmak A.B., Caton J., Papaspyridakos P., Chochlidakis K. (2020). Buccal bone thickness of maxillary anterior teeth: A systematic review and meta-analysis. J. Clin. Periodontol..

[24] Araújo M.G., Hürzeler M.B., Dias D.R., Matarazzo F. (2023). Minimal invasiveness in the alveolar ridge preservation, with or without concomitant implant placement. Periodontol. 2000.

[25] Yang S., Li Y., Liu C., Wu Y., Wan Z., Shen D. (2022). Pathogenesis and treatment of wound healing in patients with diabetes after tooth extraction. Front. Endocrinol..

[26] Jahangiri L., Devlin H., Ting K., Nishimura I. (1998). Current perspectives in residual ridge remodeling and its clinical implications: A review. J. Prosthet. Dent..

[27] Bollen A.-M., Taguchi A., Hujoel P.P., Hollender L.G. (2004). Number of teeth and residual alveolar ridge height in subjects with a history of self-reported osteoporotic fractures. Osteoporos. Int..

[28] Gupta M., Gupta M. (2015). Oral conditions in renal disorders and treatment considerations–a review for pediatric dentist. Saudi Dent. J..

[29] Saldanha J.B., Casati M.Z., Neto F.H., Sallum E.A., Nociti F.H. (2006). Smoking may affect the alveolar process dimensions and radiographic bone density in maxillary extraction sites: A prospective study in humans. J. Oral Maxillofac. Surg..

[30] Chappuis V., Engel O., Reyes M., Shahim K., Nolte L.P., Buser D. (2013). Ridge alterations post-extraction in the esthetic zone: A 3D analysis with CBCT. J. Dent. Res..

[31] Darby I., Chen S.T., Buser D. (2009). Ridge preservation techniques for implant therapy. Int. J. Oral Maxillofac. Implants.

[32] Mardas N., Trullenque-Eriksson A., MacBeth N., Petrie A., Donos N. (2015). Does ridge preservation following tooth extraction improve implant treatment outcomes: A systematic review. Clin. Oral Implants Res..

[33] MacBeth N., Trullenque-Eriksson A., Donos N., Mardas N. (2017). Hard and soft tissue changes following alveolar ridge preservation: A systematic review. Clin. Oral Implants Res..

[34] Avila-Ortiz G., Chambrone L., Vignoletti F. (2019). Effect of alveolar ridge preservation interventions following tooth extraction: A systematic review and meta-analysis. J. Clin. Periodontol..

[35] De Angelis P., Manicone P.F., Liguori M.G., D’Addona A., Ciolfi A., Cavalcanti C., Piccirillo D., Rella E. (2024). Clinical and radiographic evaluation of implant-supported single-unit crowns with cantilever extensions: A systematic review and meta-analysis. J. Prosthodont..

[36] Atieh M.A., Shah M., Hakam A., AlAli F., Aboushakra I., Alsabeeha N.H.M. (2024). Alveolar ridge preservation versus early implant placement in single non-molar sites: A systematic review and meta-analysis. Clin. Oral Implants Res..

[37] Al Yafi F., Alchawaf B., Nelson K. (2019). What Is the Optimum for Alveolar Ridge Preservation?. Dent. Clin. N. Am..

[38] Alenazi A., Alotaibi A.A., Aljaeidi Y., Alqhtani N.R. (2022). The Need for Socket Preservation: A Systematic Review. J. Med. Life.

[39] Troiano G., Zhurakivska K., Lo Muzio L., Laino L., Cicciù M., Lo Russo L. (2018). Combination of Bone Graft and Resorbable Membrane for Alveolar Ridge Preservation: A Systematic Review, Meta-Analysis, and Trial Sequential Analysis. J. Periodontol..

[40] Quisiguiña Salem C., Ruiz Delgado E., Crespo Reinoso P.A., Robalino J.J. (2023). Alveolar Ridge Preservation: A Review of Concepts and Controversies. Natl. J. Maxillofac. Surg..

[41] Suárez-López Del Amo F., Monje A. (2022). Efficacy of Biologics for Alveolar Ridge Preservation/Reconstruction and Implant Site Development: An American Academy of Periodontology Best Evidence Systematic Review. J. Periodontol..

[42] Avila-Ortiz G., Bartold P., Giannobile W., Katagiri W., Nares S., Rios H., Spagnoli D., Wikesjö U. (2016). Biologics and Cell Therapy Tissue Engineering Approaches for the Management of the Edentulous Maxilla: A Systematic Review. Int. J. Oral Maxillofac. Implant..

[43] Thoma D.S., Bienz S.P., Lim H.C., Lee W.Z., Hämmerle C.H., Jung R.E. (2020). Explorative Randomized Controlled Study Comparing Soft Tissue Thickness, Contour Changes, and Soft Tissue Handling of Two Ridge Preservation Techniques and Spontaneous Healing Two Months After Tooth Extraction. Clin. Oral Implants Res..

[44] Thalmair T., Fickl S., Schneider D., Hinze M., Wachtel H. (2013). Dimensional Alterations of Extraction Sites After Different Alveolar Ridge Preservation Techniques—A Volumetric Study. J. Clin. Periodontol..

[45] Canullo L., Del Fabbro M., Khijmatgar S., Panda S., Ravidà A., Tommasato G., Sculean A., Pesce P. (2021). Dimensional and Histomorphometric Evaluation of Biomaterials Used for Alveolar Ridge Preservation: A Systematic Review and Network Meta-Analysis. Clin. Oral Investig..

[46] Hong H.R., Chen C.Y., Kim D.M., Machtei E.E. (2019). Ridge Preservation Procedures Revisited: A Randomized Controlled Trial to Evaluate Dimensional Changes with Two Different Surgical Protocols. J. Periodontol..

[47] Avila-Ortiz G., Gubler M., Romero-Bustillos M., Nicholas C., Zimmerman M., Barwacz C. (2020). Efficacy of Alveolar Ridge Preservation: A Randomized Controlled Trial. J. Dent. Res..

[48] Domic D., Bertl K., Lang T., Pandis N., Ulm C., Stavropoulos A. (2023). Hyaluronic Acid in Tooth Extraction: A Systematic Review and Meta-Analysis of Preclinical and Clinical Trials. Clin. Oral Investig..

[49] Kim J., Song H.Y., Ben Amara H., Kyung-Rim K., Koo K. (2016). Hyaluronic Acid Improves Bone Formation in Extraction Sockets with Chronic Pathology: A Pilot Study in Dogs. J. Periodontol..

[50] James A.W., LaChaud G., Shen J., Asatrian G., Nguyen V., Zhang X., Ting K., Soo C. (2016). A Review of the Clinical Side Effects of Bone Morphogenetic Protein-2. Tissue Eng. Part B Rev..

[51] Embery G., Oliver W.M., Stanbury J.B., Purvis J.A. (1982). The Electrophoretic Detection of Acidic Glycosaminoglycans in Human Gingival Sulcus Fluid. Arch. Oral Biol..

[52] Embery G., Waddington R.J., Hall R.C., Last K.S. (2000). Connective Tissue Elements as Diagnostic Aids in Periodontology. Periodontol. 2000.

[53] Pogrel M.A., Lowe M.A., Stern R. (1996). Hyaluronan (Hyaluronic Acid) in Human Saliva. Arch. Oral Biol..

[54] Fraser J.R., Laurent T.C., Laurent U.B. (1997). Hyaluronan: Its Nature, Distribution, Functions, and Turnover. J. Intern. Med..

[55] Bertl K., Bruckmann C., Isberg P.E., Klinge B., Gotfredsen K., Stavropoulos A. (2015). Hyaluronan in Non-Surgical and Surgical Periodontal Therapy: A Systematic Review. J. Clin. Periodontol..

[56] Miglani A., Vishnani R., Reche A., Buldeo J., Wadher B. (2023). Hyaluronic Acid: Exploring Its Versatile Applications in Dentistry. Cureus.

[57] Knabe C., Adel-Khattab D., Kluk E., Struck R., Stiller M. (2017). Effect of a Particulate and a Putty-Like Tricalcium Phosphate-Based Bone-Grafting Material on Bone Formation, Volume Stability and Osteogenic Marker Expression After Bilateral Sinus Floor Augmentation in Humans. J. Funct. Biomater..

[58] Michalczyk M., Humeniuk E., Adamczuk G., Korga-Plewko A. (2022). Hyaluronic Acid as a Modern Approach in Anticancer Therapy: Review. Int. J. Mol. Sci..

[59] Lokeshwar V.B., Iida N., Bourguignon L.Y. (1996). The Cell Adhesion Molecule, GP116, Is a New CD44 Variant (Ex14/v10) Involved in Hyaluronic Acid Binding and Endothelial Cell Proliferation. J. Biol. Chem..

[60] Lesley J., Kincade P.W., Hyman R. (1993). Antibody-Induced Activation of the Hyaluronan Receptor Function of CD44 Requires Multivalent Binding by Antibody. Eur. J. Immunol..

[61] de Brito Bezerra B., Mendes Brazão M.A., de Campos M.L., Casati M.Z., Sallum E.A., Sallum A.W. (2012). Association of Hyaluronic Acid with a Collagen Scaffold May Improve Bone Healing in Critical-Size Bone Defects. Clin. Oral Implants Res..

[62] Zhai P., Peng X., Li B., Liu Y., Sun H., Li X. (2020). The Application of Hyaluronic Acid in Bone Regeneration. Int. J. Biol. Macromol..

[63] Park D., Kim H., Kim K., Lee Y.-S., Choe J., Hahn J.-H., Lee H., Jeon J., Choi C., Kim Y.-M. (2012). Hyaluronic Acid Promotes Angiogenesis by Inducing RHAMM-TGFbeta Receptor Interaction via CD44-PKCdelta. Mol. Cells.

[64] Bonifacio M.A., Cassano A., Vincenti A., Vinella A., Dell’olio F., Favia G., Mariggiò M.A. (2023). In Vitro Evaluation of the Effects of Hyaluronic Acid and an Aminoacidic Pool on Human Osteoblasts. Biomedicines.

[65] Ariyoshi W., Okinaga T., Knudson C.B., Knudson W., Nishihara T. (2014). High Molecular Weight Hyaluronic Acid Regulates Osteoclast Formation by Inhibiting Receptor Activator of NF-κB Ligand Through Rho Kinase. Osteoarthr. Cartil..

[66] Kloss F.R., Kau T., Heimes D., Kämmerer P.W., Kloss-Brandstätter A. (2024). Enhanced Alveolar Ridge Preservation with Hyaluronic Acid-Enriched Allografts: A Comparative Study of Granular Allografts with and Without Hyaluronic Acid Addition. Int. J. Implant Dent..

[67] Pirnazar P., Wolinsky L., Nachnani S., Haake S., Pilloni A., Bernard G.W. (1999). Bacteriostatic Effects of Hyaluronic Acid. J. Periodontol..

[68] Dahiya P., Kamal R. (2013). Hyaluronic Acid: A Boon in Periodontal Therapy. N. Am. J. Med. Sci..

[69] Rodrigues S.V., Acharya A.B., Bhadbhade S., Thakur S.L. (2010). Hyaluronan-Containing Mouthwash as an Adjunctive Plaque-Control Agent. Oral Health Prev. Dent..

[70] Eick S., Renatus A., Heinicke M., Pfister W., Stratul S.I., Jentsch H. (2013). Hyaluronic Acid as an Adjunct After Scaling and Root Planing: A Prospective Randomized Clinical Trial. J. Periodontol..

[71] Xu Y., Hofling K., Fimmers R., Frentzen M., Jervoe-Storm P.M. (2004). Clinical and Microbiological Effects of Topical Subgingival Application of Hyaluronic Acid Gel Adjunctive to Scaling and Root Planing in the Treatment of Chronic Periodontitis. J. Periodontol..

[72] Pistorius A., Martin M., Willershausen B., Rockmann P. (2005). The Clinical Application of Hyaluronic Acid in Gingivitis Therapy. Quintessence Int..

[73] Sapna N., Vandana K.L. (2011). Evaluation of Hyaluronan Gel (Gengigel) as a Topical Applicant in the Treatment of Gingivitis. J. Investig. Clin. Dent..

[74] Shirakata Y., Imafuji T., Nakamura T., Kawakami Y., Shinohara Y., Noguchi K., Pilloni A., Sculean A. (2021). Periodontal Wound Healing/Regeneration of Two-Wall Intrabony Defects Following Reconstructive Surgery with Cross-Linked Hyaluronic Acid-Gel with or Without a Collagen Matrix: A Preclinical Study in Dogs. Quintessence Int..

[75] Shirakata Y., Nakamura T., Kawakami Y., Imafuji T., Shinohara Y., Noguchi K., Sculean A. (2021). Healing of Buccal Gingival Recessions Following Treatment with Coronally Advanced Flap Alone or Combined with a Cross-Linked Hyaluronic Acid Gel. J. Clin. Periodontol..

[76] Alcântara C.E.P., Castro M.A.A., de Noronha M.S., Martins-Junior P.A., Mendes R.d.M., Caliari M.V., Mesquita R.A., Ferreira A.J. (2018). Hyaluronic Acid Accelerates Bone Repair in Human Dental Sockets: A Randomized Triple-Blind Clinical Trial. Braz. Oral Res..

[77] Lorenz J., Barbeck M., Kirkpatrick C.J., Sader R., Lerner H., Ghanaati S. (2018). Injectable Bone Substitute Material on the Basis of Beta-TCP and Hyaluronan Achieves Complete Bone Regeneration While Undergoing Nearly Complete Degradation. Int. J. Oral Maxillofac. Implants.

[78] Cocero N., Ruggiero T., Pezzana A., Bezzi M., Carossa S. (2019). Efficacy of Sodium Hyaluronate and Synthetic Amino Acids in Post-Extractive Sockets in Patients with Liver Failure: Split-Mouth Study. J. Biol. Regul. Homeost. Agents.

[79] Marin S., Popovic-Pejicic S., Radosevic-Caric B., Trtic N., Tatic Z., Selakovic S. (2020). Hyaluronic Acid Treatment Outcome on the Post-Extraction Wound Healing in Patients with Poorly Controlled Type 2 Diabetes: A Randomized Controlled Split-Mouth Study. Med. Oral Patol. Oral Cir. Bucal.

[80] Eeckhout C., Ackerman J., Gilbert M., Cosyn J. (2022). A Randomized Controlled Trial Evaluating Hyaluronic Acid Gel as a Wound Healing Agent in Alveolar Ridge Preservation. J. Clin. Periodontol..

[81] Cosola S., Oldoini G., Boccuzzi M., Giammarinaro E., Genovesi A., Covani U., Marconcini S. (2022). Amino Acid-Enriched Formula for the Post-Operative Care of Extraction Sockets Evaluated by 3-D Intraoral Scanning. Int. J. Environ. Res. Public Health.

[82] Husseini B., Friedmann A., Wak R., Ghosn N., Khoury G., El Ghoul T., Abboud C.K., Younes R. (2023). Clinical and Radiographic Assessment of Cross-Linked Hyaluronic Acid Addition in Demineralized Bovine Bone-Based Alveolar Ridge Preservation: A Human Randomized Split-Mouth Pilot Study. J. Stomatol. Oral Maxillofac. Surg..

[83] Abaza G., Abdel Gaber H.K., Afifi N.S., Adel-Khattab D. (2024). Injectable Platelet-Rich Fibrin Versus Hyaluronic Acid with Bovine Derived Xenograft for Alveolar Ridge Preservation: A Randomized Controlled Clinical Trial with Histomorphometric Analysis. Clin. Implant. Dent. Relat. Res..

[84] Kim J.J., Ben Amara H., Park J.-c., Kim S., Kim T.-I., Seol Y.-J., Lee Y.-M., Ku Y., Rhyu I.-C., Koo K.-T. (2019). Biomodification of Compromised Extraction Sockets Using Hyaluronic Acid and rhBMP-2: An Experimental Study in Dogs. J. Periodontol..

[85] Lee J., Chu S., Ben Amara H., Song H., Son M., Lee J., Kim H., Koo K., Rhyu I. (2021). The Effects of Hyaluronic Acid and Deproteinized Bovine Bone Mineral with 10% Collagen for Ridge Preservation in Compromised Extraction Sockets. J. Periodontol..

[86] Misch C.E., Perel M.L., Wang H.-L., Sammartino G., Galindo-Moreno P., Trisi P., Steigmann M., Rebaudi A., Palti A., Pikos M.A. (2008). Implant Success, Survival, and Failure: The International Congress of Oral Implantologists (ICOI) Pisa Consensus Conference. Implant Dent..

[87] Mansour A., Acharya A.B., Alliot C., Eid N., Badran Z., Kareem Y., Rahman B. (2024). Hyaluronic Acid in Dentoalveolar Regeneration: Biological Rationale and Clinical Applications. J. Oral Biol. Craniofac. Res..

[88] dos Santos Canellas J.V., Medeiros PJ D.A., da Silva Figueredo C.M., Fischer R.G., Ritto F.G. (2019). Which Is the Best Choice After Tooth Extraction: Immediate Implant Placement or Delayed Placement with Alveolar Ridge Preservation? A Systematic Review and Meta-Analysis. J. Craniomaxillofac. Surg..

[89] Nazir M.A., AlGhamdi L., AlKadi M., AlBeajan N., AlRashoudi L., AlHussan M. (2018). The Burden of Diabetes, Its Oral Complications, and Their Prevention and Management. Open Access Maced. J. Med. Sci..

[90] Thoma D.S., Buranawat B., Hämmerle C.H., Held U., Jung R.E. (2014). Efficacy of Soft Tissue Augmentation Around Dental Implants and in Partially Edentulous Areas: A Systematic Review. J. Clin. Periodontol..

[91] Clementini M., Castelluzzo W., Ciaravino V., Agostinelli A., Vignoletti F., De Sanctis M. (2020). Impact of Treatment Modality After Tooth Extraction on Soft Tissue Dimensional Changes: A Randomized Controlled Trial. Clin. Oral Implants Res..

